# A simple protocol for isolating mouse lung endothelial cells

**DOI:** 10.1038/s41598-018-37130-4

**Published:** 2019-02-06

**Authors:** Jinping Wang, Niu Niu, Suowen Xu, Zheng Gen Jin

**Affiliations:** 1 Department of Medicine, Aab Cardiovascular Research Institute, University of Rochester, Rochester, NY 14623 USA; 2 Department of Pharmacy, Shenzhen Second People’s Hospital, Shenzhen, 518035 China

## Abstract

Endothelial dysfunction is the common molecular basis of multiple human diseases, such as atherosclerosis, diabetes, hypertension, and acute lung injury. Therefore, primary isolation of high-purity endothelial cells (ECs) is crucial to study the mechanisms of endothelial function and disease pathogenesis. Mouse lung ECs (MLECs) are widely used in vascular biology and lung cell biology studies such as pulmonary inflammation, angiogenesis, vessel permeability, leukocyte/EC interaction, nitric oxide production, and mechanotransduction. Thus, in this paper, we describe a simple, and reproducible protocol for the isolation and culture of MLECs from adult mice using collagenase I-based enzymatic digestion, followed by sequential sorting with PECAM1 (also known as CD31)- and ICAM2 (also known as CD102)-coated microbeads. The morphology of isolated MLECs were observed with phase contrast microscope. MLECs were authenticated by CD31 immunoblotting, and immunofluorescent staining of established EC markers VE-cadherin and von Willebrand factor (vWF). Cultured MLECs also showed functional characteristics of ECs, evidenced by DiI-oxLDL uptake assay and THP-1 monocyte adhesion assay. Finally, we used MLECs from endothelium-specific enhancer of zeste homolog 2 (EZH2) knockout mice to show the general applicability of our protocol. To conclude, we describe here a simple and reproducible protocol to isolate highly pure and functional ECs from adult mouse lungs. Isolation of ECs from genetically engineered mice is important for downstream phenotypic, genetic, or proteomic studies.

## Introduction

Endothelial cells (ECs) are one of the most important cell types in the circulatory system, which exist in all blood vessels of the heart, lung, brain, liver, and many other tissues. ECs are the gate-keeper of cardiovascular, metabolic and pulmonary health by serving as natural barrier of circulating blood and human body as well as a platform for substance exchange^1,2^. Endothelial dysfunction is the common mechanism of multiple human diseases, such as atherosclerosis, diabetes, hypertension, and lung injury^3,4^. Primary culture of ECs is an important tool to dissect the role of endothelial genes in endothelial dysfunction-associated disorders. Currently, several types of ECs, such as HUVECs (human umbilical vein endothelial cells), HAECs (human aortic endothelial cells), HCAECs (human coronary artery endothelial cells), HLMECs (human lung microvascular endothelial cells), BAECs (bovine aortic endothelial cells), and SAECs (swine aortic endothelial cells) are widely used in cardiovascular research^5^. Due to the ease of genetic engineering and other advantages, mouse is one of the most frequently used species for study cardiovascular diseases^6^. The isolation of ECs from mice has been successfully used in phenotypic, and genetic studies characterizing endothelial genes in human diseases^7,8^. There are several protocols describing the isolation of ECs, from different tissues/organs/vascular beds, such as MAECs (mouse aortic endothelial cells)^9,10^, immortalized MAECs (iMAECs)^5^, MLECs (mouse lung endothelial cells)^11–13^, MBMECs (mouse brain microvascular endothelial cells)^14^, MCMEC (mouse cardiac microvascular endothelial cells)^15^, and MLSECs (mouse liver sinusoidal endothelial cell)^16^. These different tissue-resident ECs could have common vascular functions, as well as some specialized functions. Among EC culture from different tissues, MLECs and MAECs are commonly used (Table 1). Difference of these protocols lies in the use of adult mice versus neonatal mice; different digestion time of the lung (mostly 45–60 min); and the use of dynabeads versus flow cytometry for the sorting^12^. Due to the small size of mice (compared with other large experimental animals), and limited amount of tissue sources, several mice need to be pooled for isolating ECs from mice in a routine procedure.

In this report, we developed an easy-to-follow, step-by-step protocol to isolate highly pure and functionally competent ECs from lung tissues using single adult mice (without pooling several mice). We also discussed important features/modifications of this protocol and provide troubleshooting suggestions, with an aim to ensuring the successful isolation of MLECs. This protocol can be applied to study multiple aspects of endothelial function and dysfunction *in vitro*.

## Results

### Morphology of adult MLECs

**Figure 1:**
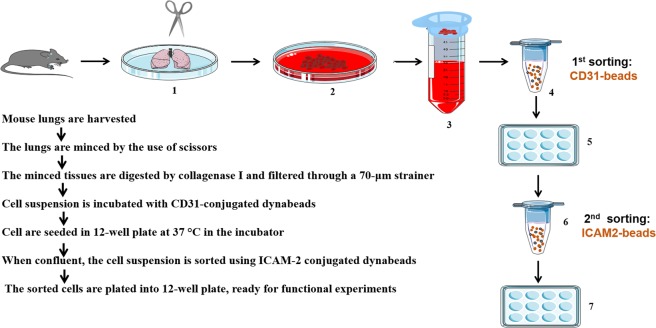
Diagram of microbeads-based protocol for the isolation of MLECs.

**Figure 2:**
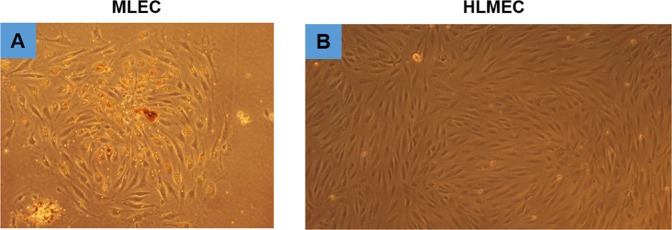
Morphology of cultured MLECs as compared to normal adult Human Lung Microvascular Endothelial Cells. (**A**) Image of cultured mouse lung endothelial cells (MLECs), original magnificationX10, n = 3. (**B**) Image of cultured Human Lung Microvascular Endothelial Cells (HLMECs, Sigma-Aldrich, # 540-05 A), original magnificationX10, n = 3.

The step-by-step procedure of MLECs isolation and culture is shown in Fig. 1. In brief, mice lung tissues were harvested, minced, and digested in 3 mg/ml Collagenase I for 45 min, before filtering through 70 µm cell strainer. Cells were spinned down, and incubated with CD31-conjugated dynabeads for the first sorting. When cells reach confluence, second sorting was performed using ICAM2-conjugated dynabeads. Finally, cells were seeded in 12 well plates till experiments without further culture. Using this protocol, we observed that morphology of cultured MLECs at confluent stage resembles that of HLMECs, indicating it is a reliable *in vitro* system to analyze endothelial function or dysfunction (Fig. 2).

### Identification of adult MLECs

**Figure 3:**
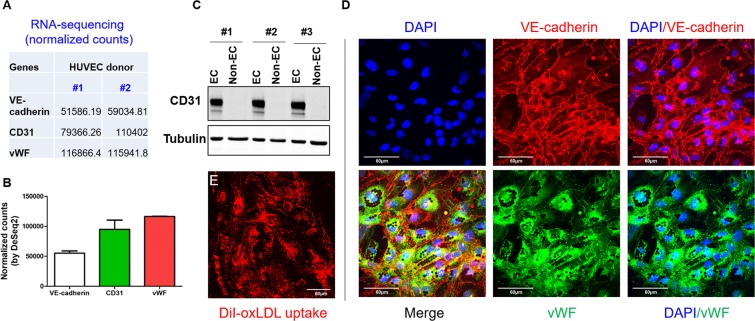
Identification of cultured MLECs by western blot and confocal microscopy. (**A**,**B**) RNA-sequencing data mined from published literature^19^ shows expression abundance of three EC marker genes, VE-cadherin (also known as CDH5), CD31 (also known as PECAM1), and vWF. Data shown are normalized counts from two different donors of HUVECs. (**C**) CD31 expression of EC fraction (CD31^+^; ICAM2^+^) and non-EC (CD31^−^; ICAM2^−^) fraction after second bead sorting, n = 3 different mice. (**D**) VE-cadherin (red) and vWF (green) staining of cultured MLEC by confocal microscopy, n = 4, scale bar = 60 um. (**E**), DiI-oxLDL uptake (red) by cultured MLECs, n = 5, scale bar = 60 um.

Several EC markers are commonly used for EC identification, including VE-cadherin (gene name: CDH5), CD31 (gene name: PECAM1), and von Willebrand factor (vWF)^17^. Some studies also used CD146 as an EC marker^18^. Mining of published RNA-seq database^19^ indicates that, in HUVECs, gene expression pattern of these three markers is: vWF > CD31 > VE-cadherin (Fig. 3A,B). To further validate the purity of cultured MLECs, the expression of CD31 in both MLECs after 2^nd^ sorting (EC fraction, CD31^+^; ICAM2^+^) and non-bound ECs (CD31^−^; ICAM2^−^ fraction) we compared. We observed CD31 expression only in EC fraction, however, CD31 is absent from non-EC fraction, suggesting the majority of ECs has been pulled down by magnetic beads (Fig. 3C). Our confocal microscope data also support that >99% of cultured MLECs were VE-cadherin^+^ and vWF^+^ (Fig. 3D). DiI-oxidized LDL (DiI-oxLDL) uptake assay (Fig. 3E) indicated that cultured MLECs have engulfing capacity of oxLDL.

### Responses of isolated MLECs to inflammatory cytokines and to attract monocytes

**Figure 4:**
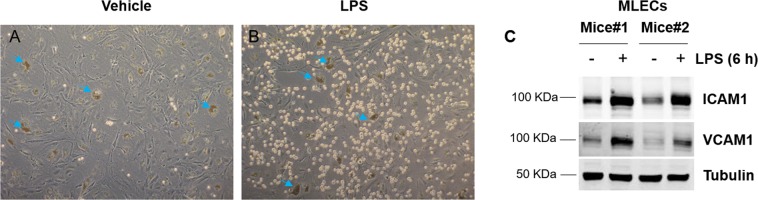
Cultured MLECs respond to inflammatory treatment. (**A**,**B**) MLECs were treated with vehicle (**A**, saline) or LPS (**B**) for 6 h before monocyte adhesion assay was performed as described in method section, n = 4. The brown dots are dynabeads. (**C**) MELCs were treated with vehicle or LPS for 6 h before whole cell lysate was collected for western blot analysis to detect pro-adhesive molecules ICAM1 and VCAM1 protein expression, n = 2 different mice.

The inflammatory response is a critical component of host defense. Multiple stimuli, such as TNFα, can trigger leukocyte adhesion to activated ECs^20^. Our cultured ECs show increased monocyte adhesion and adhesion molecule (ICAM1 and VCAM1) expression (Fig. 4A,B,C). Therefore, cultured MLECs using this protocol, can be used for *in vitro* leukocyte adhesion assay.

### Application of MLECs to validate endothelium specific EZH2 knockout mice

**Figure 5:**
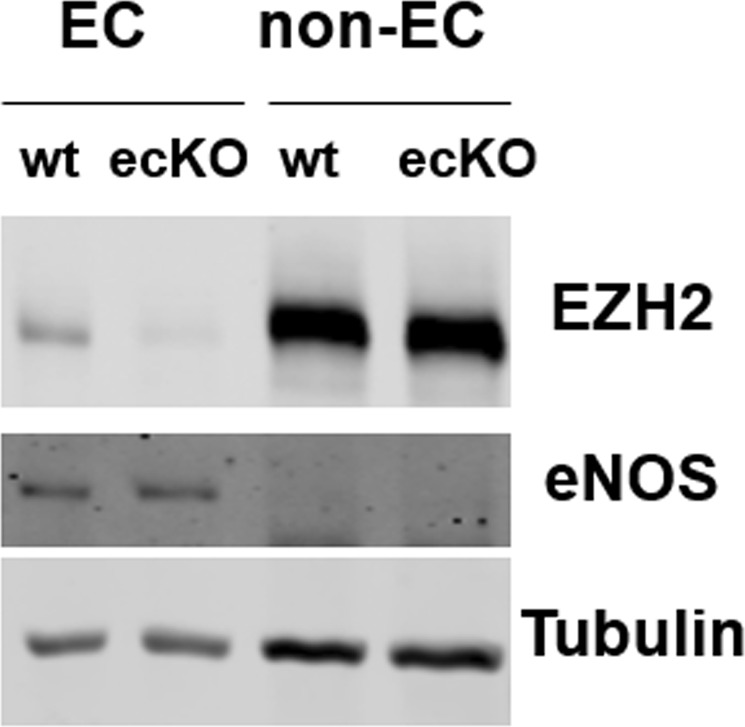
Culture of MLECs to validate endothelium specific deletion of EZH2 in EZH2-ecKO mice. MLECs were isolated from EZH2^flox/flox^ (wt) and endothelium-specific EZH2 knockout mice (ecKO) mice. Whole cell lysate from EC fraction (CD31^+^; ICAM2^+^) and non-EC (CD31^−^; ICAM2^−^) fraction were collected for western blot to detect EZH2 protein expression using Tubulin as the loading control. Endothelial nitric oxide synthase (eNOS) was only expressed in the EC fraction. Representative images from 2 pairs of mice were shown.

Finally, to prove the utility of our protocol, we used cultured MLECs to validate the successful deletion of EZH2 in EC specific EZH2 knockout (EZH2-ecKO; VE-cadherin-Cre+; EZH2^flox/flox^) mice. As shown in Fig. 5, compared with control mice (EZH2^flox/flox^), EZH2 protein expression is significantly reduced in ECs from EZH2-ecKO mice. However, EZH2 expression in non-ECs fraction remain unaffected.

## Discussion

Cardiovascular disease ranked #1 in all causes of human morbidity and mortality^21–25^. Endothelial dysfunction, a constellation of multiple critical events, such as hyperpermeability, leukocyte adhesion, impaired nitric oxide production, represents a major mechanism for the development of cardiovascular diseases^4^. ECs, innermost cell type lining the surfaces of blood vessels, serve as a natural barrier between circulating blood and vessel component. It also plays critical roles in multiple pathophysiological processes, such as angiogenesis, vascular permeability, inflammation, hemostasis, and mechanotransduction^3^. While, EC dysfunction may contribute significantly to the development of vascular diseases such as thrombosis, and atherosclerosis^3^. The primary culture of ECs is critical to study endothelial dysfunction.

Although primary or immortalized MLECs are commercially available, considering the high cost of purchasing and great tendency to experience cell transition, primary isolation of MLECs remain very common in routine laboratory practice. Several protocols have been described in the literature to isolate ECs from mouse lungs^5,11,12^. Most isolation of MLECs used collagenase I with or without dispase for the digestion of lung tissues^11^. There are several other protocols available to isolate ECs from other mouse tissues, such as mouse aorta^17^, liver^16^, etc., using Matrigel-based aortic explants, enzymatic digestion followed by beads selection or cell sorting by flow cytometry. Several commonly used markers of endothelial cells, such as CD31, eNOS, VE-cadherin, vWF, and CD146, are frequently used for validating the EC identity^17,18^. Most MAEC culture protocols used Matrigel as the base matrix for isolation (Table 1). Due to the fact that Matrigel induces capillary-like tube formation in ECs and angiogenesis. Matrigel may create a pro-angiogenic environment which stimulates cell proliferation and migration, potentially altering the phenotype of primary cells^5^. Alternative matrix, such as type I collagen gel, has been used to isolate ECs with less effects on cellular phenotype^5^. In our protocol, we successfully isolated MLECs from adult mice without using Matrigel or collagen gel during isolation processes. The endothelial identity was confirmed by cell morphology, expression of EC marker proteins, CD31, VE-cadherin, and vWF by western blot and immunofluorescent staining. Due to some EC marker proteins do exist in a small fraction of other cell types, the combinatorial use of >2 markers is suggested to eliminate the issue of non-specificity. The cultured MELCs can uptake DiI-oxLDL and respond to LPS stimulation and attract more monocyte adhesion upon LPS stimulation. This technique also proved successful in isolating ECs from endothelium-specific EZH2 knockout mice, indicating the application potential in confirming tissue specific knockout of endothelial genes as well as allowing for analysis of endothelial transcriptomic changes after genetic/pharmacological interventions.

**Table 1:** Exemplified protocols for the isolation of ECs from mouse lung and aorta.

Type of EC (tissue)	Age	Method of isolation	Sorting method	Reference
MLECs (lung)	3–4 month old	Collagenase A digestion of minced tissue (37 °C, 1 h)	Double magnetic bead sort (ICAM2)	^38^
	Neonatal mice	Collagenase I/dispase digestion of minced tissue (37 °C, 45 min)	Double magnetic bead sort (CD31/ICAM2)	^11^
	Neonatal mice	collagenase A digestion of minced tissue (37 °C, 30 min)	FACS or magnetic beads (ICAM2)	^12^
	NA	Collagenase I digestion of minced tissue (37 °C, 45 min)	Double magnetic bead sort (CD31 for both sorting)	^39^
	7–8-week-old	Enzyme Mix plus MACS™ dissociator (37 °C, 30 min)	Magnetic cell sorting using CD146 Microbeads (Miltenyi Biotec)	^40^
MAECs (aorta)	Adult	Lumen digestion (Collagenase II) (37 °C, 45 min)	NA	^10^
	NA	Matrigel + aortic ring explant	NA	^9, 17^
	4-week-old	Type I Collagen gel + aortic ring explant	NA	^5^

Abbreviations: CD31, cluster of differentiation 31; FACS, Fluorescence activated cell sorting; ICAM2, intracellular adhesion molecule 2; MLECs, mouse lung endothelial cells; MAEC, mouse aortic endothelial cells.

### Comparison of current protocol with established protocols

The procedure presented here is a modified protocol, based on several previous published protocols^11–13^. Our protocol adopts similar two-step sorting using CD31, and ICAM2-coated dynabeads to isolated MLECs. We optimized the isolation protocol by reducing the amount of beads, reducing the number of mice for isolation (from pooled mice to a single mice), using adult mice for isolation, and seeding cells in relatively high density to allow for high endothelial proliferative capacity. Due to the heterogeneity of ECs, a combination of several endothelial markers is also an advantage for isolating highly pure ECs. We also provide our experience in the isolation and cell culture, as well as troubleshooting in the protocol. By using this protocol, we can consistently and reliably obtain pure populations of ECs from adult mouse lungs. This approach of isolating MLECs has been recently applied to study endothelial function (such as leukocyte adhesion and inflammation) in our laboratory for several studies^26,27^.

### Factors/considerations for MLECs culture protocol

There are several technical aspects of this protocol that are important for the MLECs isolation and culture from mouse lungs.

1. Using serum-free DMEM media (rather than PBS) to dissolve collagenase I increase cell viability, attachment, and final yield.
2. The degree of mincing lung tissues and complete dissociation of lungs into single cells determines final cell yield. Also, underdigestion (<45 min) tend to cause insufficient tissue dissociation and cell isolation, and overdigestion of lung tissues (>60 min) tend to reduce cell viability. Complete dissociation of lungs into single cells is a prerequisite for reliable cell separation and cell analysis. Complete dissociation can be achieved by combinatorial use of enzymatic digestion with mechanical dissociation by pipetting or using the gentle MACS^TM^ Dissociator (Miltenyi Biotec). Gentle tissue dissociation is important for preserving the cell surface epitopes.
3. Always use freshly isolated cells (P1) for cell culture experiments (usually immediately for experiments after second bead sorting). This is because primarily isolated MLECs are very easy to differentiate to fibroblast-like cells (mesenchymal) when cultured for a long time period^5^ despite the fact these cells retain ECs markers (such as VE-cadherin) and functions (DiI-oxLDL uptake).
4. It is critical to seed cells in high-density in small volume dish (12-well plate in our case) to avoid cell senescence. Specifically, if cells were seeded in large dishes, such as 100 mm dishes, cells take very long time to be confluent, easy to cause cell senescence. It is plausible that high-density cultures will ensure endothelial cells to grow faster by secreting multiple pro-growing factors in an autocrine and paracrine manner.
5. The amount of beads used in each sorting. The beads we are using are Dynabeads® Sheep Anti-Rat IgG. The beads are 4.5 µm superparamagnetic beads conjugated with polyclonal sheep anti-rat IgG (https://www.thermofisher.com/order/catalog/product/11035?SID=srch-srp-11035). Since beads ingestion is a stress for endothelial cells, and during the whole culture period, endothelial cells cannot digest or exclude all beads from inside the cells. Therefore, to minimize the effect of beads on cell behaviors/functions, for isolating MLECs from 1 adult mouse, we recommend using 25 µl coated CD31 dynabeads to the first isolation, and 20 µl coated ICAM2 dynabeads for the second sorting. The amount of beads can be increased or decreased proportionally according number of mice harvested. Although freshly prepared coated beads are recommended for each isolation, we did not see significant loss of binding activity of MLECs if the coated dynabeads are stored at 4 degree within a week. Binding activity is compromised after long time storage. The beads inside the cells may have unwanted functions on endothelial cells, and cell-conjugated beads can be cleaved by enzyme if necessary. Beads have autofluorescence under confocal microscopy and appear like small and round hollow spots.
6. We noticed that vWF staining of MLECs from a culture of pooled mice is not homogenous even though almost all cells were VE-cadherin positive, suggesting that endothelial cells from mice have heterogeneity of vWF expression.
7. This protocol can be applied for isolating ECs from mouse livers and neonatal mouse lungs without modification (data not shown).
8. Pooled lungs *vs* single lung: MLECs can be harvested from single mice or pooled several mice. In our protocol, MLECs were isolated from single mice, and the exact number of mice is the “N” number for each experiment. If mice lungs (2 or 3) were pooled, to reach statistical significance, at least 6 to 9 mice per each group has to be used. Therefore, our protocol significantly reduces the mouse number to isolate primary ECs, complying with the ‘3Rs’ principles (Replacement, Reduction and Refinement) of animal research.
9. Considerations on re-genotyping. For experiments involving genetically engineered mice, re-genotyping before isolation is recommended as mouse ear tags fall or mouse ear punches close sometimes. Otherwise, label each dish with mouse ID, and re-label genotype if previous wrong genotyping information is identified. Re-genotyping before isolation is more critical if mouse lungs are pooled in each group.
10. Cell passage: Due to this nature of transformation, mouse ECs are normally used in experiments after sorting without further passaging. However, the immortalized MAECs can be passaged for several times^5^. The sorted cells are then plated and split 1:2 at each passage to maintain a high density of the cells to avoid the development of senescence^12^. In most cases, cultured MLECs can be passaged several times. We do observe changes of cell morphology after extended culture for several passages, which could potentially limit its application in endothelial mesenchymal transition.
11. Double sorting: We noticed that one time CD31 beads selection is always not enough to obtain pure EC population, as we can observe many cells existing in the CD31-negative fraction.
12. Using two or more markers for EC identification. Given the heterogeneity and specialized functions of ECs in vascular beds or tissues, it is better to use a combination of two or more antibodies for validating the EC identity. We also have to realize that data obtained from cultured MLECs, need to validated in primary cultured human ECs and, more importantly, *in vivo*.
13. Contamination: Thus, in sorting the cells in this way, one is also likely to separate ECs associated with other cells, including fibroblasts. Common contaminants of EC culture are fibroblasts, smooth muscle cells, and some resident macrophages^17^. We have to point out that, endothelial cells are one cell type that engulf DiI-oxLDL, other cell types, such as fibroblasts and macrophages, can also uptake DiI-oxLDL, although with different binding capacity^12^.
14. Age of mice for isolating MLECs: In our experience, we found that the isolated MLECs did not proliferate well after freezing and thawing. As whole lungs were used to isolate MLECs, we can anticipate that MLECs prepared using this protocol is a combination of ECs from both the pulmonary and bronchial circulation^12^. We noticed that MLECs from mice at neonatal stage or younger age proliferate faster than aged mice, which result in lower yields of ECs. In our hands, lung from each individual mouse can result in good yields and high purity.
15. Beads washing. To avoid cell death during washing steps, all steps must be performed gently and 0.1% BSA was included in the washing solution. During the first sorting of ECs from mouse lung, since it has been observed that a cluster of ECs together with some of tissue matrix was pulldown by beads, great care must be taken to avoid vacuum system for the aspiration. Pipet aspiration is recommended.
16. Binding buffer for beads: In our experiences, we observe DPBS performs better than serum-free DMEM in beads binding reactions. The possible reason could be that some buffer component could potentially affect beads binding.
17. Coating matrix: In our protocol, we use 1% gelatin for coating the culture dishes, and we did not compare other coating matrixes, such as collagen I and fibronectin. A previous study has shown that MEAC, have a better morphology and grow faster when coated with collagen I, compared with gelatin-coating or non-coating plates^10^. The selection of different coating material could affect the signaling pathways in functional assays, such as mechanotransduction.
18. Choice of pulldown antibodies: As stipulated in the user manual of dynabeads, the choice of primary antibody is the most important factor for successful cell isolation. Some antibodies may show reduced antigen-binding efficiency when coated onto beads, despite good performance of this antibody in other assays (such as western blot). Adding aggregated IgG to block Fc-receptors prior to adding the primary antibodies can be used to block non-specific binding of cells.

### Applications of MLECs

1. Studying endothelial function: previous studies have shown that cultured MLECs can be used for multiple assays for assessing endothelial function, such as angiogenesis^28^, vascular tone^29^, and inflammation^30^. Here, we used LPS induced inflammatory response and monocyte adhesion as a model to test the responsiveness of cultured cells.
2. Validation of endothelium-specific knockout or transgenic mice as well as performing phenotypic, genetic, or proteomic studies. Genetically engineered mice are frequently used in vascular biology studies. In particular, endothelium-specific conditional knockout mice (using traditional Cre-LoxP or CRISPR/Cas9 technology) or endothelium-specific transgenic mice are of great value in investigate the cell-specific functions of targeted molecules in regulating endothelial cell function, cardiovascular, metabolic, and pulmonary disease. For example, Zhang *et al*. have used MLECs to confirm the deletion of LKB1 from endothelium specific LKB1 knockout mice^31^. A recent study have used MLECs from EC-specific progerin transgenic mice to dissect the mechanism of aging associated endothelial dysfunction and cardiovascular impairment^32^. In our study, we used cultured MLECs to confirm the successful deletion of EZH2 in EZH2-ecKO mice. Isolation of endothelial cells from these genetically engineered mice is also important for downstream phenotypic, genetic, or proteomic studies.
3. Cultured MLECs can be used for next generation RNA-sequencing: RNA-sequencing is an emerging new technology that enables investigators to understand the gene regulation at the genome-wide scale^33^. MLECs can be used to evaluate the effect of gene deletion or overexpression in endothelial transcriptome.

### Important notes on MLECs culture

1. Change in cell morphology: Cultured MLECs are very easy to be transformed in terms of cell morphology^5^, which could potentially limit their potential in some functional assays, such as endothelial mesenchymal transition assays, since these cells display an elongated morphology (like HLMECs), unlike the cobblestone morphology of HUVECs.
2. Injury during lung mincing and non-specific binding of coated dynabeads: mincing steps could potentially result in patho-physiological trauma to MLECs which could potentially influence cell viability^12^. Second, nonspecific binding of the beads to non-endothelial cells has been noticed in another study^12^. Optimizing the amount of beads addition is critical, which pulldown ECs as much as possible, and limit the non-specific binding of beads with other contaminating cell types.

### Study limitations

1. In this study, we used human THP-1 monocytes in the monocyte adhesion assay under static conditions, due to the species difference and hemodynamic forces *in vivo*, it is better to use isolated mouse peripheral blood monocytes or mouse monocytic cell lines (such as WEHI-274.1, ATCC® CRL-1679™) to assess genetic/pharmacological manipulation on adhesive property of monocytes to ECs under flow conditions.
2. In this study, we used western blot and immunofluorescence to confirm the purity and characterize responsiveness of cultured ECs. It is better to use alternative methods, such as flow cytometry, which is a more sensitive and more quantitative than immunofluorescence and western blot, to calculate the exact purity of ECs and endothelial responsiveness to pro-inflammatory treatment.

## Conclusion

We provide a detailed, simple, and reproducible procedure to isolate MLECs from single adult mice. Our protocol has important applications in vascular biology and lung cell biology studies, such as for vascular permeability, leukocyte adhesion, and vascular homeostasis. Our protocol is also instructive to other researchers in developing extended protocols for isolating ECs from other mouse tissues, such as liver and heart. The culture of MLECs will provide opportunities to investigate the precise roles of targeted endothelial genes in growing number of conditional knockout and transgenic mouse lines as well as probing the cellular and molecular mechanism of cardiovascular diseases.

## Methods

### Mice

The Institutional Animal Care of University of Rochester Medical Center (URMC) approved all animal care procedures. All experiments were performed in accordance with the relevant guidelines and regulations by URMC. Wild-type C57BL/6 J mice are purchased from Jackson Laboratories). To generate endothelial cell specific knockout mice deficient of EZH2, female EZH2^flox/flox^ mice (gifted by Jia Guo, University of Rochester) were mated with male VE-cadherin-Cre mice (The Jackson Laboratory, Stock No. 006137^34^. Then, male VE-cadherin-Cre^+^; EZH2^flox/+^ mice were cross bred with EZH2^flox/flox^ females to generate endothelial cell specific EZH2 knockout mice (EZH2-ecKO) (VE-cadherin-Cre^+^; EZH2^flox/flox^).

### Cell culture

1. Human Lung Microvascular Endothelial Cells (HLMECs), Sigma, catalog number: 540-05 A;
2. Human monocyte cells line THP-1, gifted by Y. Cai (Harvard Medical School, MA).

### Chemicals and Reagents

1. Type I Collagenase, Thermo Fisher Scientific, catalog number: 17100017;
2. DMEM media, Corning, catalog number: 10-013-CV;
3. Endothelial cells growth supplement (ECGS), Cell Applications Inc, catalog number: 212-GS;
4. Penicillin/Streptomycin, Thermo Fisher Scientific, catalog number: 15140122;
5. DPBS, Thermo Fisher Scientific, catalog number: 14190250;
6. Rat anti-mouse CD31 antibody (BD Bioscience, catalog number: 553370); for beads coating
7. Rat anti-mouse ICAM2 antibody (BD Bioscience, catalog number: 553326); for beads coating
8. Rat anti-mouse VE-cadherin antibody (BD Bioscience, catalog number: 555289); for western blot
9. Rabbit anti-human vWF antibody (DAKO, catalog number: A0082); for immunofluorescence
10. 30% BSA in DPBS (Sigma, catalog number: A9576);
11. DiI-ox-LDL (Alfa Aesar, catalog number: J64164);
12. Mouse monoclonal anti-Tubulin (Sigma Aldrich, catalog number: T6074); for western blot
13. VCAM1 (R&D Biosystems, catalog number: AF796); for western blot
14. ICAM1 (R&D Biosystems, catalog number: AF643); for western blot
15. Rabbit anti-mouse CD31 (Abcam, catalog number: ab124432); for western blot
16. Rabbit EZH2 antibody (Cell Signaling Technologies, catalog number: 5246); this antibody recognize both mouse and human EZH2, for western blot
17. Sheep anti-Rat IgG dynabeads (Thermo Fisher Scientific, catalog number: #11035); for beads coating
18. 70 µm Cell Strainer (Corning, catalog number: 352350);
19. Trypsin/EDTA Solution (TE): Thermo Fisher, catalog number: R00110;
20. Trypsin Neutralizer Solution (TN): Thermo Fisher, catalog number: R002100;
21. Lipopolysaccharides (LPS) from *Escherichia coli* O111:B4, Sigma-Aldrich, catalog number: L4391

### Equipment

1. Confocal microscopy, Olympus, Model: IX81
2. 12-Tube Magnetic Separation Rack, Cell Signaling Technologies, catalog number: 14654
3. Centrifuges, Beckman Coulter, Model Allegra X-15R
4. VWR Rocking platform, VWR, Model 200

### Recipes

1. Isolation solution: DMEM + 20% FBS + Penicillin/Streptomycin
2. Digestion solution: 0.22 µm filtered 3 mg/mL collagenase I, in DMEM
3. Beads washing solution: DPBS + 0.1%BAS + Penicillin/Streptomycin
4. Complete culture medium: DMEM + 20% FBS + 1X ECGS + 100 µg/ml heparin* + Penicillin/Streptomycin

*The purpose of heparin is to inhibit the growth of smooth muscle cells^17^.

### Step-by-step protocol of isolation and culture of MLECs

#### Preparation of antibody-coated dynabeads

The day before cell harvest,100 µl dynabeads was incubated with 10 µl CD31 antibody (CD31-coated beads, for first sorting) and 10 µl ICAM2 antibody (ICAM2-coated beads, for second sorting), respectively, under constant stirring at 4 degree overnight. Then, the suspension was placed on magnetic rack and the supernatant as aspirated, and the bound beads were washed three times with beads washing solution. After each wash, the coated beads are placed on the magnetic rack for 1 min. Supernatant was carefully aspirated without disturbing the beads. After the final wash, the antibody-coated dynabeads were suspended with same volume of beads washing solution (100 µl) and stored at 4 degree for use.

#### Mouse lung tissue dissociation and digestion

2–3 month-old C57BL/6 J mice were used for MLECs isolation. Mice were anaesthetized with ketamine (100 mg/kg) /xylazine (10 mg/kg) and sacrificed. Mouse lungs were harvested immediately (without perfusion) from freshly euthanized adult mice and stored in 8 ml isolation buffer in 60 mm dish or 15 ml conical tubes. Immersed tissues were placed on ice before mincing with autoclaved scissors. Scissors were changed when switching to the next mice. Mice lungs were placed in an empty 60 mm cell culture dishes using forceps under a sterile cell culture hood, and drain the media around the lung using 1 ml pipetman. The lung was shredded using scissors, about 100 times, and digested with 8 ml of collagenase I at 37 °C for 45 min. Culture suspensions were stirred every 15 min, to ensure that all the pieces of lung tissue was well dislodged and do not clump as tissue trunks. This procedure enables a complete digestion of the lung tissue and dispersal of resident cells.

#### MLECs sorting using CD31-coated beads (1^st^ sorting)

Tissue suspension was passed through 5 ml syringe with 20 G cannula attached and triturate clumps into a single cell suspension, at least 12 times. The minced tissue was filtered through a 70-μm cell strainer seated on top of a 50 ml Falcon tube pre-placed with isolation buffer (which contain serum to stop digestion). Cell suspension was mixed and centrifuged at 1200 rpm, 4 °C for 8 min. Supernatant was carefully removed and cell pellets was resuspended in 1 ml beads washing solution, and transferred to 1 ml Eppendorf tube. Routinely, 25 μl CD31 coated dynabeads was added into the suspension, and incubated at room temperature for 15 min with robust shaking on the rocking platform. After that, suspension was washed five to eight times with isolation media, and resuspended in 1 ml complete culture media, and plated in 0.1% gelatin-coated 12-well plate at 37 °C in a cell culture incubator with 5% CO_2_. The next day, the culture media was aspirated, and new complete media was added. Cell morphology was examined under a phase contrast microscope till cell reach confluence. After first sorting, CD31-dynabeads were endocytosed into endothelial cells. Due to the limited number of CD31^+^ cells in first sorting, beads were not removed/cleaved. Beads can be diluted out after 2–3 passages.

#### MLECs sorting using ICAM2-coated beads (2^nd^ sorting)

When cultured cells reach 90% confluent, cells were detached with 0.15 ml TE solution, followed by adding 0.15 ml TN solution to stop trypsinization. 0.9 ml complete media was then added. Cell suspension (1.2 ml) was spinned down at room temperature at 1200 rpm for 5 min. Supernatant was aspirated, and cells were resuspended in 1 ml beads washing solution, and 20 µl ICAM2-coated beads washing solution was added. Cell suspensions were mixed on the rocking platform as described above, and cell pellets were suspended in 1 ml complete culture media, and then seeded into 12-well plate. Culture media was changed every 48 h. Cells are ready for experiments when reach confluent monolayer. In most cases, cells were immediately used for experiments without further passage after second sorting.

### Uptake of DiI-oxLDL

MLECs were seeded on 0.1% gelatin-coated glass-bottom dish overnight. The next day, cells were incubated in serum-free DMEM containing 10 µg/ml DiI-oxLDL at 37 °C for 4 h. After washing with PBS three times to remove unbound free DiI-oxLDL, cells were fixed with 4% paraformaldehyde (PFA, in PBS) for 15 min at room temperature with gentle shaking on the shaking platform. Images were observed with confocal microscopy. Due to the penetration process by 0.1% Triton X-100 quenches DiI fluorescence, cells were not penetrated and not co-stained with DAPI.

### LPS induced inflammation in MLECs

Monocyte adhesion assay was performed as previously described^20^. In brief, MLECs seeded in 12-well plates were randomly divided into two groups: (1) control group: cells treated with vehicle (saline); (2) LPS group: cells were treated with LPS (1 µg/mL) for 6 h. THP-1 monocytes were added to each well and incubated for an additional 30 min. The non-adherent cells were aspirated and the monolayer was gently washed with PBS three times. Images at three random optical fields were taken. In some cases, cells were lysed in lysis buffer and western blot was used to detect the expression of two well-established pro-adhesive molecules, VCAM1 and ICAM1.

### Confocal microscopy

ECs have several well-established marker proteins, which include CD31, VE-cadherin, and vWF. The combination of two or more marker proteins is important for validating the identity of cultured endothelial cells. To this end, immunofluorescent studies were performed as previously described^20^. MLECs were cultured on 0.1% gelatin-coated chamber slides, and washed in PBS three times, and then permeabilized with 0.1% Triton X100 for 15 min at room temperature. Cells were blocked in 10% goat serum and incubated with rat anti mouse VE-cadherin and rabbit anti-mouse vWF primary antibodies, followed by incubations with goat anti-rat and goat anti-mouse secondary antibodies at room temperature for 1 h. DAPI was used to counterstain cell nuclei.

### Western blotting

After cell rinsing with ice-cold PBS, whole cell lysate was prepared and western blot was prepared as previously described^35–37^. The protein content of each sample was determined by Bio-Rad DC assays. Aliquots of cell lysate (20 μg of protein) were then resolved by size on 10% SDS-polyacrylamide gels and subsequently transferred to a NC membrane. The membrane was blocked with LICOR blocking buffer, and incubated with a primary antibody overnight at 4 °C, followed by incubation with an LICOR secondary antibodies for 30 min at room temperature in darkness. Membranes were washed in 1XTBST, and subject to LICOR imaging.

## Data Availability

The datasets generated during and/or analysed during the current study are available from the corresponding author on reasonable request.
